# Signal averaged ECG in patients with early repolarization

**DOI:** 10.1002/joa3.12523

**Published:** 2021-03-05

**Authors:** Mani Hassanzadeh, Ehsan Mardani, Alireza Hosseinpour, Zahra Mehdipour Namdar, Shahab Shahrzad, Amir Aslani

**Affiliations:** ^1^ Shiraz University of Medical Sciences Shiraz Iran; ^2^ Cardiovascular Research Center Shiraz University of Medical Sciences Shiraz Iran; ^3^ Fars Heart Foundation Kowsar Hospital Shiraz Iran

**Keywords:** early repolarization, J point elevation, signal averaged electrocardiography

## Abstract

**Background:**

Early repolarization (ER) pattern is diagnosed when the J‐point is elevated on the patient's electrocardiogram. The aim of this study was to evaluate signal‐averaged electrocardiography (SAECG) in patients with ER pattern.

**Methods:**

Subjects were divided into three groups: 1‐patients with normal ECG pattern (control group); 2‐patients with J‐point elevation in the inferior leads; and 3‐patients with J‐point elevation in non‐inferior leads.

**Results:**

The mean filtered QRS duration in groups with J‐point elevation in inferior leads and non‐inferior leads and in the control, was 86.4 ± 23.4 msec, 84.8 ± 26.6 msec, and 85.8 ± 24.8 msec, respectively, indicating no significant difference across the three groups. The mean duration of terminal QRS < 40µV was 21.2 ± 4.2 msec, 22.8 ± 4.6 msec, and 23.1 ± 4.5 msec in the mentioned groups, respectively, without a significant difference between the groups. Additionally, the mean root‐mean‐square voltage of terminal 40 msec was 34.5 ± 8.3 µV, 35.3 ± 8.6µV, and 35.7 ± 9.2 µV in patients with increased J‐point in inferior leads, non‐inferior leads, and the control group, respectively, showing no difference between the groups.

**Conclusion:**

In conclusion, we found that parameters in SAECG did not have any significant difference between patients with ER pattern and healthy individuals. Moreover, we concluded that SAECG cannot distinguish the patients with elevated J‐point in inferior leads from non‐inferior leads. Overall, SAECG does not appear to be a reliable diagnostic tool for the assessment of ER pattern.

## Graphical abstract

In this study, we evaluated signal‐averaged electrocardiography (SAECG) in patients with early ER pattern and we found that parameters in SAECG didn't have any significant difference between patients with ER pattern and healthy individuals.

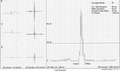

## 1 INTRODUCTION

Early repolarization (ER) is defined as the J‐point elevation detected on the patient's electrocardiogram (ECG). Despite the previous thoughts, arrhythmias and sudden cardiac death may occur in ER, where high amplitude J‐point elevation is seen on the ECG. The J‐point elevation in ER could be horizontal and/or downsloping ST segments, locating in the inferior and/or lateral leads. The prevalence of ER varies from 3% to 24% in different reports.^1^, ^2^ There might be two main categories of clinical manifestations for ER considering the ECG results, including: 1‐ syncope and survivors after cardiac arrest and 2‐ ECG pattern in asymptomatic patients randomly diagnosed with ER.^3^, ^4^

Aiming to improve the noninvasive manners to identify the patients who are susceptible to reentrant ventricular tachycardia, signals averaged ECG (SAECG) has been developed over the recent years, which is a modality to evaluate and detect the occult impairment of ventricular activation.^5^, ^6^, ^7^, ^8^

The aim of this study was to evaluate the SAECG in patients with ER pattern and detect any possible relation between the SAECG parameters and ER pattern.

## 2 MATERIALS AND METHODS

### 2.1 Study population

A total of 38 consecutive patients (age 33.1 ± 7.7 years) under follow‐up for ER and 20 healthy male subjects (age 28.3 ± 6.8 years) were included in the study. All patients underwent a 12‐lead ECG and transthoracic echocardiography. Early repolarization pattern is diagnosed on the surface ECG by the presence of J‐point elevation ≥1 mm in ≥2 contiguous inferior and/or lateral leads of a standard 12‐lead ECG. This study received approval from the University Institutional Review Board and written informed consent was obtained from the subjects.

### 2.2 Early repolarization syndrome

Early repolarization syndrome is diagnosed by the presence of J‐point elevation ≥1 mm in ≥2 contiguous inferior and/or lateral leads of a standard 12‐lead ECG in a patient resuscitated from otherwise unexplained ventricular fibrillation/ polymorphic ventricular tachycardia.

### 2.3 Measurement of SAECG

SAECG was recorded by resting 12‐Lead during sinus rhythm with bipolar X, Y, and Z lead (Biomedical Systems rev.5.0.3). The QRS complexes were amplified, digitized, and averaged (50‐100 beats). In each assessment, three parameters were computed:

1. Duration of the filtered QRS complex (fQRSd),
2. Root mean square of amplitude in terminal 40 ms (RMS40),
3. Duration of low amplitude signal (LAS40).

Abnormalities were detected if the filtered QRS complex was more than 114 ms, the square of the terminal signal was lower than 20 µV or a low amplitude signal longer than 38 ms Patients were considered as having late potentials (LPs) if they had abnormalities in at least two SAECG indices.

### 2.4 Exclusion criteria

Patients with the following criteria were excluded from the study: history of coronary artery disease, abnormal LV systolic function (EF < 50%), significant valvular heart disease, bundle branch block (QRS > 120 msec), and patients who use anti‐arrhythmic drugs.

### 2.5 Statistical analysis

Continuous data were presented as mean ± standard deviation. Normality of data was analyzed using the Kolmogorov‐Smirnoff test. The one‐way analysis of variance (ANOVA) was used to determine whether there are any statistically significant differences between the means of the three groups. *P* values less than .05 were considered statistically significant. For the statistical analysis, the statistical software SPSS version 13 for windows (SPSS Inc, Chicago, IL) was used.

## 3 RESULTS

A total of 38 patients (38 males) including 14 patients with elevated J‐point in inferior leads [aged 29.2 ± 5.9 years] and 24 patients with evident J‐point elevation on other leads of ECG [aged 27.8 ± 5.5 years] were recruited. A group of 20 healthy subjects (20 males) without any change in ECG pattern with the mean age of 28.3 ± 6.8 years were studied as a control group. The patients were matched to the control group with respect to age and gender. The mean filtered QRS complex duration in the group with elevated J‐point in inferior leads, non‐inferior leads, and control group was 86.4 ± 23.4 msec, 84.8 ± 26.6 msec, and 85.8 ± 24.8 msec, respectively, indicating no significant differences between the three groups (Tables 1 and 2). Moreover, the mean duration of terminal QRS < 40µV was not significantly different among the control group and patients with J‐point elevation (Tables 1 and 2). The root‐mean‐square voltage of the terminal 40 msec was also not significantly different between the patients with J‐point elevation in inferior leads (34.5 ± 8.3 µV), the ones with J‐point elevation in non‐inferior leads (35.3 ± 8.6µV), and the control group (35.7 ± 9.2 µV) (Tables 1 and 2).

**TABLE 1:** SAECG parameters in the group with early repolarization in inferior leads (A), non‐inferior leads (B), and control group (C)

	ER in Inferior leads [n = 14] (A)	ER in non‐inferior leads[n = 24] (B)	Control Group [n = 20] (C)	*P*‐value (A vs. C)	*P*‐value (B vs. C)
Filtered QRS duration (ms)	86.4 ± 23.4	84.8 ± 26.6	85.8 ± 24.8	.65	.72
Duration of terminal QRS < 40µV (ms)	21.2 ± 4.2	22.8 ± 4.6	23.1 ± 4.5	.63	.68
Root‐mean‐square voltage of terminal 40msec (μv)	34.5 ± 8.3	35.3 ± 8.6	35.7 ± 9.2	.80	.86

Abbreviation: ER, early repolarization.

**TABLE 2:** The one‐way analysis of variance (ANOVA) in three groups with early repolarization

Variable	Groups	*Sig. [between groups]
Filtered QRS duration (ms)	ER in Inferior Leads ER in Non‐Inferior Leads Control Group	0.44
Duration of terminal QRS < 40µV (ms)	ER in Inferior Leads ER in Non‐Inferior Leads Control Group	0.23
Root‐mean‐square voltage of terminal 40msec (μv)	ER in Inferior Leads ER in Non‐Inferior Leads Control Group	0.83

* The mean difference is significant at the 0.05 level.

### 3.1 Early repolarization syndrome

**FIGURE 1:**
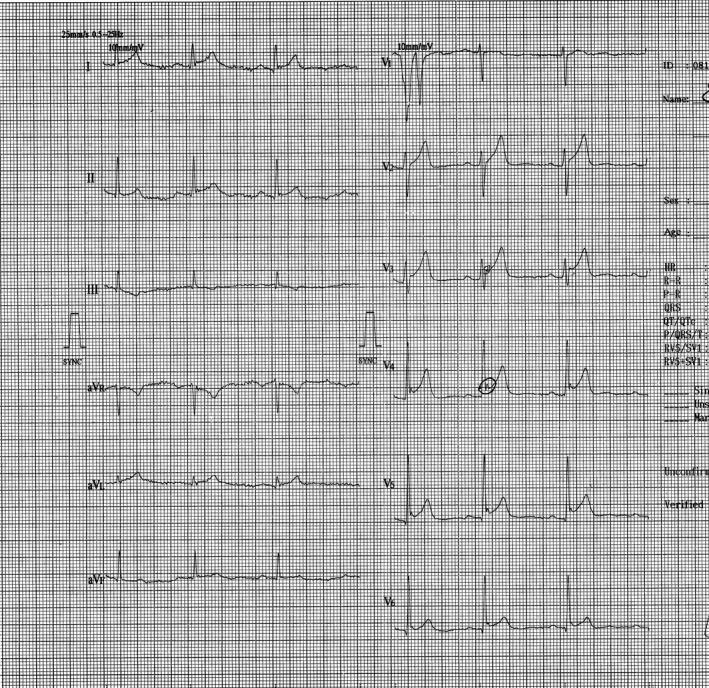
Shows the 12‐lead surface ECG of a patient with otherwise unexplained ventricular fibrillation. J‐point elevation is seen in lead V4, V5, and V6

**FIGURE 2:**
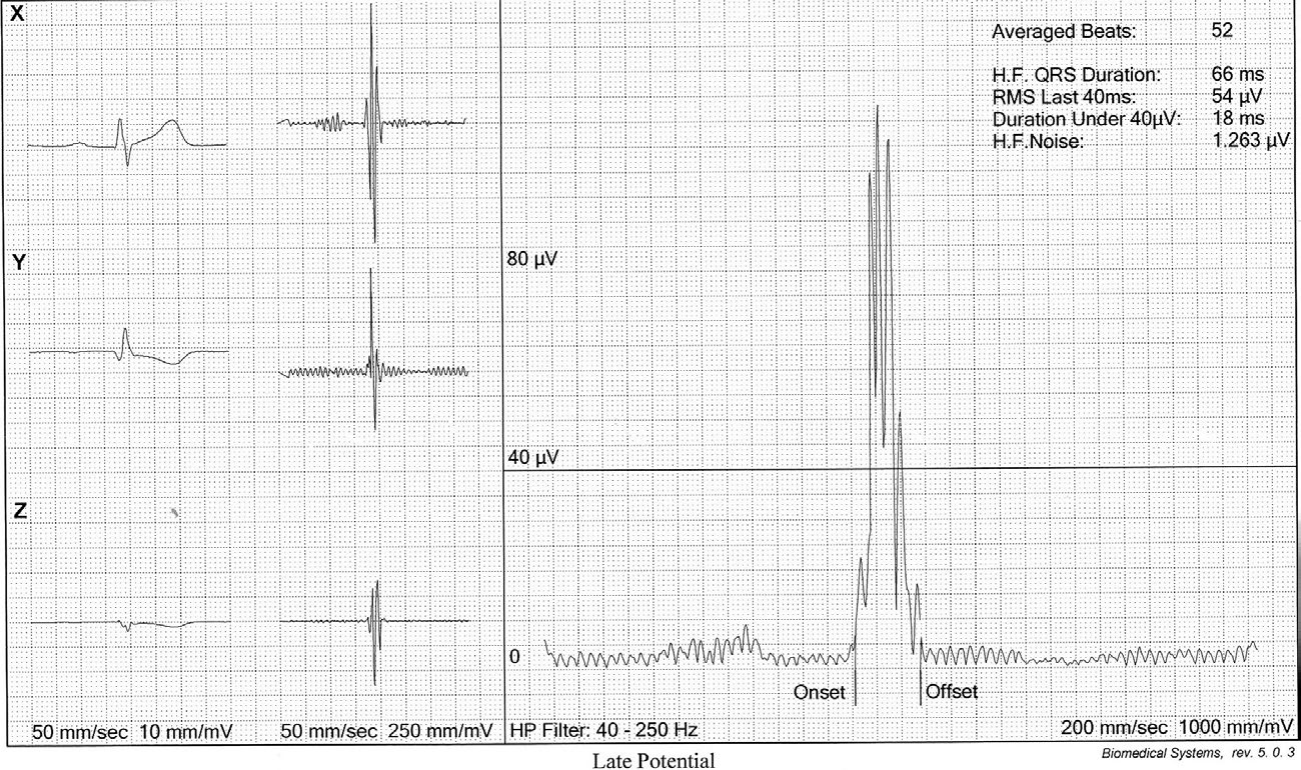
Shows SAECG parameters of the patient mentioned in Figure 1

Three patients with J‐point elevation in surface ECG had a previous history of aborted sudden cardiac death (SCD). These three patients were resuscitated from otherwise unexplained ventricular fibrillation (ER syndrome). Implantable cardioverter‐defibrillator (ICD) was implanted for all the three patients for the secondary prevention of SCD. The values of SAECG parameters of these three patients are demonstrated in Table 3. SAECG parameters of these three patients revealed no evidence of late potentials. Surface ECG and SAECG of patient#1 with aborted SCD are illustrated in Figures 1 and 2.

**TABLE 3:** Baseline characteristics and SAECG parameters in patients with ER syndrome

	Gender	Age	Presentation	Therapeutic Strategy	fQRSd	LAS40	RMS40	J wave amplitude	J wave localization
Patient#1	male	36	Aborted SCD	ICD implantation	66	18	54	3 mm	V4‐V5‐V6
Patient#2	male	31	Aborted SCD	ICD implantation	88	21	34	2 mm	II, III, aVf
Patient#3	male	34	Aborted SCD	ICD implantation	88	23	35	2 mm	II, III, aVf

Abbreviations: ER, early repolarization; ICD, implantable cardioverter defibrillator; SCD, sudden cardiac death.

### 3.2 J‐Wave amplitude and SAECG parameters

Patients were divided into two groups according to J‐wave amplitude:

- Group‐1: Patients with J‐wave amplitude <0.2 mV,
- Group‐2: Patients with J‐wave amplitude ≥0.2 mV

Table 4 shows SAECG parameters in the two groups (according to the J‐wave amplitude). There were no significant differences between SAECG parameters and J‐wave amplitude.

**TABLE 4:** J‐wave amplitude and SAECG parameters

	J‐wave amplitude < 0.2 mV (n = 31)	J‐wave amplitude ≥ 0.2 mV (n = 7)	*P*‐value
Filtered QRS duration (ms)	84.5 ± 21.3	87.3 ± 22.6	.55
Duration of terminal QRS < 40µV (ms)	23.1 ± 3.3	22.1 ± 4.2	.63
Root‐mean‐square voltage of terminal 40msec (μv)	33.2 ± 7.2	32.2 ± 5.4	.51

## 4 DISCUSSION

The traditional benign nature of the early repolarization (ER) pattern has been a matter of debate in the last few years. An ER pattern is noticed in up to 24% of the overall normal population. Recently, studies have pointed out the possible malignant nature of ER pattern in leading to polymorphic ventricular tachycardia (VT) and ventricular fibrillation (VF) and subsequently, sudden cardiac death (SCD). An ER pattern is defined as an increased J‐point level >0.1 mV in two or more contiguous inferior and/or lateral leads excluding leads V1‐V3 in a standard 12‐lead ECG. This condition is considered as ER syndrome in a patient survived from a VT or VF without any previous cardiovascular disease.^2^, ^9^, ^10^, ^11^, ^12^ Tikkanen et al^13^ investigated the probable correlation between early repolarization and mortality from cardiac causes in a cohort study. They found out that the presence of J‐point elevation >0.1 mV is associated with cardiac and arrhythmic related deaths (relative risk >1) and a significant J‐point elevation >0.2 mV is significantly linked with mortality from cardiac causes (relative risk >2). The abovementioned findings emphasize the importance of finding new tools in order to facilitate the diagnosis and early assessment of ER pattern and its possible association with the likelihood of ventricular dysfunction resulting in VT and VF.

Iwakami et al^8^ aimed to differentiate malignant from benign ER pattern by performing magnetocardiography (MCG) on patients diagnosed with ER pattern resuscitated from VF (ERP‐VF(+)) and another group of patients with ER pattern without any history of VF (ERP‐VF(‐)). The results demonstrated that MCG parameters were remarkably different in the two groups and they concluded that MCG can be a useful technique in distinguishing malignant from benign ER pattern with high sensitivity.

SA‐ECG is a non‐invasive diagnostic electrocardiographic technique that averages multiple electric cardiac signals facilitating the detection of subtle abnormalities in the surface ECG. This method generally identifies high frequency and low‐intensity signals localized at the end of the QRS complex, which is defined as late ventricular potentials (VLPs). The appearance of VLPs in SAECG may indicate myocardial scar tissue in ventricles leading to delayed ventricular activation.^14^ SAECG is being used in identifying the patients with a higher risk of ventricular dysfunction and reentrant ventricular tachyarrhythmia and hence, detecting the patients with a predisposition toward SCD.^15^ In a study by Tahara et al^16^ the filtered QRS duration was measured in SAECG in patients with ventricular dyssynchrony. The results suggested that filtered QRS duration is a reliable measure in SAECG and it is related to ventricular dyssynchrony. As a result, one can conclude that SAECG can be considered as a tool in assessing conditions with ventricular dysfunction.

In this study we intended to measure SAECG parameters including QRS complex duration, duration of terminal QRS with <40 micro voltages, and root‐mean‐square voltage of the terminal 40 msec using a 12‐lead resting ECG, which is the gold standard and the most reliable tool in ECG monitoring, to find any possible association between ER pattern and SAECG markers. Notably, none of the aforementioned markers in SAECG showed any significant difference between patients with an ER pattern and control group. Also, we studied the SAECG parameters in patients with ERS (diagnosed with a J‐point elevation in the 12‐lead ECG and a past medical history of aborted SCD) and found no significant change in parameters of SAECG compared to healthy subjects. In a study by Soliman et al,^17^ they tended to acquire a better understanding of any association between ER and markers of ventricular arrhythmogenesis (defined as VLPs in SAECG) using a three‐channel recorder Holter monitoring in a population of patients referred for palpitation with no history of previous unexplained SCD and they found no significant correlation between ER and measures of SAECG. In our study, we measured the parameters of SAECG obtaining a resting 12‐lead ECG, which is the gold standard and the most reliable tool in ECG monitoring. We also included patients with ER syndrome with a previous history of aborted SCD and measured the parameters in them. Soliman and colleagues obtained the SAECG parameters using an ambulatory ECG recording (Holter) to measure the parameters of SAECG in patients with ER pattern and they had no patients with the history of aborted SCD (ER syndrome) in their study.

Dahdah et al^18^ compared the SAECG measurements in children with Kawasaki disease and history of aortic aneurysm. They found out that the patients with a history of persistent aortic aneurysm had a markedly lower terminal 40 msec root mean square than the groups with late and early resolution of aortic aneurysm; however, we did not find any correlation between the root‐mean‐square voltage of the terminal 40 msec in SAECG and presence of J‐point elevation in our study. Furthermore, we could not find any significant difference in SAECG parameters between patients with J‐point elevation in inferior leads and the ones with elevated J‐point in non‐inferior leads.

## 5 CONCLUSION

In conclusion, we found that parameters in SAECG including filtered QRS duration according to millisecond, duration of terminal QRS <40 micro voltages, the root‐mean‐square voltage of terminal 40 milliseconds did not have any significant difference between patients with ER pattern and healthy individuals. Moreover, we concluded that SAECG cannot distinguish the patients with elevated J‐point in inferior leads from non‐inferior leads. Overall, SAECG does not appear to be a reliable diagnostic tool for the assessment of ER pattern.

## CONFLICT OF INTEREST

None.

## Data Availability

The data underlying this article will be shared at reasonable request to the corresponding author.
